# Occurrence of per- and polyfluoroalkyl substances (PFAS) and their precursors in biosolids across 15 U.S. states

**DOI:** 10.1007/s11356-026-38066-3

**Published:** 2026-07-21

**Authors:** Xiayan Ye, Amith Sadananda Maroli, Santhoshi Chitthaluri, Erin M. Driver, Rolf U. Halden, Arjun K. Venkatesan

**Affiliations:** 1 Cleveland Clinic Research, Cleveland Clinic, Cleveland, OH 44195 USA; 2 Department of Environmental Medicine and Climate Science, Icahn School of Medicine at Mount Sinai, New York, NY 10029 USA; 3 Department of Civil and Environmental Engineering, New Jersey Institute of Technology, Newark, NJ 07102 USA; 4 Center for Environmental Health Engineering, The Biodesign Institute, Arizona State University, Tempe, AZ 85281 USA; 5 Department of Geology, University of Georgia, Athens, GA 30602 USA

**Keywords:** Biosolids, EPA 1633, TOP assay, PFOA, PFOS, PFAS, Sludge

## Abstract

The US Environmental Protection Agency (EPA) draft sewage sludge risk assessment indicates that land application of biosolids containing as little as 1 ng/g of perfluorooctanoate (PFOA) or perfluorooctane sulfonate (PFOS) may exceed human health risk thresholds. This study provides updated measurements of per- and polyfluoroalkyl substances (PFAS) in US biosolids. Twenty-seven biosolid samples from 15 states were analyzed using EPA Method 1633 and the total oxidizable precursor (TOP) assay. Dominant PFAS included 5:3 fluorotelomer carboxylic acid (FTCA) (70.2 ± 84.7 ng/g), 7:3 FTCA (23.7 ± 43.7 ng/g), and PFOS (22.1 ± 45.2 ng/g), with total PFAS ranging from 2 to 807 ng/g, showing no correlation with sewer-shed population or industrial inputs. At one facility, PFAS increased from 17.3 ng/g in primary sludge to 150 ng/g in centrifuge cake, consistent with precursor transformation. TOP assay did not increase total PFAS levels but shifted the profile, with perfluoroalkyl carboxylic acids increasing and FTCAs and other precursors decreasing, likely reflecting incomplete transformation of precursors during wastewater and sludge treatment. These findings suggest that regulated PFAS will increase following land application due to transformation of precursors over time. Compared to the 2001 national sewage sludge survey samples, PFOA and PFOS declined by > 80%, yet remain at least one order of magnitude above 1 ng/g. Current study supports EPA’s conclusion that land application of biosolids continues to pose potential human health risks.

**Supplementary Information:**

The online version contains supplementary material available at 10.1007/s11356-026-38066-3.

## Introduction

PFAS are a class of > 15,000 synthetic chemicals ubiquitous in the environment, sourced from widespread industrial and agricultural uses (Brase et al. 2021; CompTox 2025). Due to the high energy required for dissociation of carbon-fluorine bonds, PFAS tend to persist in the environment and cannot be easily broken down by conventional treatment methods or via natural attenuation (Hakeem et al. 2024). Studies have reported detectable PFAS levels in water, air, soil, wildlife, and humans globally (Nakayama et al. 2019; De Silva et al. 2021; Criswell et al. 2024). Even low-level PFAS exposures have been associated with adverse health effects (Domingo and Nadal 2019; Fenton et al. 2021; DeLuca et al. 2022).

PFAS are frequently detected in wastewater treatment plants (WWTPs), groundwater, and stormwater (Thompson et al. 2022). Conventional WWTPs are not designed to remove these compounds, and hence effluents are considered a point source of PFAS to the environment (Abunada et al. 2020). During passage through WWTPs, PFAS in wastewater tend to partition into sewage sludge due to their adsorptive properties (Venkatesan and Halden 2013; Schaefer et al. 2022). Processed and treated sewage sludge or biosolids are applied on land as a fertilizer and thus can serve as an additional source of PFAS contamination in the environment (Venkatesan and Halden 2014a; Bolan et al. 2021; Johnson 2022). The US EPA released its Draft Risk Assessment for perfluorooctanoic acid (PFOA) and perfluorooctane sulfonic acid (PFOS) in sewage sludge in January 2025, where the assessment evaluated human health risks from land application, surface disposal, and incineration of biosolids (EPA 2025). In some modeled scenarios, applying biosolids containing only 1 ppb (ng/g) of PFOA or PFOS exceeded EPA risk thresholds by several orders of magnitude. Elevated risks were observed across all three disposal pathways. The models were not conservative, assuming low-end concentrations and median exposures, and did not account for multiple PFAS, combined exposures, or additional sources such as consumer products and drinking water (EPA 2025). Hence, determining the occurrence and levels of PFAS in biosolids is critical to assess the risk of land application of biosolids but also challenging due to biosolids’ complex matrix. Furthermore, the lack of a standardized method until now for biosolids (EPA Method 1633 A 2024) has led to the reporting of variable PFAS levels in past studies.

The first US nationwide survey of PFAS in biosolids analyzed archived samples collected during the 2001 EPA National Sewage Sludge Program and detected 13 PFAS with PFOS showing the highest concentration (403 ± 127 ng/g dry weight (dw)), followed by PFOA (34 ± 22 ng/g dw) and PFDA 26 ± 20 ng/g dw) (Venkatesan and Halden 2013). Since 2001, individual PFAS have been subjected to various industrial production phase-outs and regulatory actions. Perfluorohexane sulfonate (PFHxS), PFOS, perfluorodecane sulfonate (PFDS), and related precursors were phased out of production in the early 2000 s and PFOA and related precursors were phased out of production in the early 2010 s in North America (ITRC 2020). Most of the previous studies have found that long-chain PFAS like PFOS tend to be the dominant compound detected in biosolids (Higgins et al. 2005; Munoz et al. 2022), but short-chain PFAS such as perfluorohexanoic acid (PFHxA) are also frequently detected (Gravesen et al. 2023). More recently, Link et al. (2024) collected biosolids from 190 WWTPs in Michigan, USA, and analyzed for 24–29 PFAS. The authors reported total detected PFAS concentrations ranging from 1 to 3200 ng/g dw and perfluoroalkyl sulfonates (PFSAs) comprising 71% of the total detectable PFAS concentration (Link et al. 2024). Schaefer et al. (2023) collected biosolids from 38 WWTPs located in 23 US states and reported the mean of sums of detected PFAS to be 160 ± 46 ng/g dw, where authors adapted the biosolid extraction method from a previously published protocol (Sepulvado et al. 2011; Lee et al. 2014; Shojaei et al. 2022). US EPA recently finalized Method 1633, which is an isotope dilution liquid chromatography tandem mass spectrometry (LC–MS/MS) method that quantifies 40 PFAS in complex sample matrices including biosolids. A recent study reported significant variation in PFAS concentration when analyzing split samples in different laboratories employing EPA Method 1633 (Oza et al. 2025a), suggesting the heterogeneity of the matrix could be the major factor.

In WWTPs, terminal PFAS levels in the effluent have been repeatedly observed at higher concentrations than those in the influent. This is mainly due to precursor transformation during wastewater treatment (Schultz et al. 2006; Guerra et al. 2014; Filipovic and Berger 2015; Chen et al. 2018; Lakshminarasimman et al. 2021). PFAS precursor degradation (or transformation) can potentially lead to the formation of other PFAS subclasses that can either exist in the effluent or, more importantly, partition into the sludge (Higgins et al. 2005; Sinclair and Kannan 2006). This is important because the presence of high organic matter in solids can influence the chemistry of the precursor and terminal PFAS. For example, studies have shown that in the presence of high organic matter, losses of PFAS such as FTOHs from water to air are decreased (Liu et al. 2007; Wang et al. 2011). Thus, the sludges produced by WWTPs may represent a reservoir of high concentrations of unknown PFAS precursors and of potentially persistent and toxic chemicals posing both human and ecological risks, particularly if these materials are reused in agriculture (Chaney et al. 1996). The total oxidizable precursor (TOP) assay is a key analytical tool used to quantify the potential of PFAS precursors to degrade into terminal PFAS compounds (Houtz and Sedlak 2012). This assay facilitates the identification of PFAS that may not be detected by conventional methods, providing a more comprehensive understanding of the total PFAS burden in biosolids. A recent study by Kim Lazcano et al. (2020) reported a two- to three-fold increase in perfluoroalkyl acids (PFAAs) following oxidation, indicating the presence of substantial amounts of transformable precursors.

The primary goal of the present study was to provide an updated baseline level of PFAS and their precursors in US biosolids utilizing EPA Method 1633 and the TOP assay. The specific objectives of the study were to (i) assess the method performance utilizing a reference material of sludge from National Institute of Standards and Technology (NIST), (ii) identify dominant compounds across different facilities and PFAS composition, and (iii) assess the presence of oxidizable precursors of PFAS.

## Materials and methods

### Sampling

Sludge samples were collected from different wastewater and biosolid processing facilities across 15 states in the USA. A total of 27 grab samples were stored at −20 °C at Arizona State University, and aliquots were shipped in high-density polyethylene bottles to Stony Brook University, for extraction and analysis using EPA Method 1633 and the TOP assay. The samples were collected between 2014 and 2020, represented a variety of matrices, including dewatered composite sludge, centrifuge cake, Class B biosolids (treated sewage sludge that meets EPA pathogen-reduction requirements for land application but remains subject to site-use restrictions), dewatered sludge, and primary and waste-activated sludge. Additional details on facility locations and sample characteristics are provided in Table SI-1 in the supplementary material.

### Sample analysis

#### Chemicals and materials

PFAS native and isotopically labeled standards were purchased as mixture solutions from Wellington Laboratories (Guelph, ON). Mixed standard solutions of the analytes and their surrogates and internal standards were prepared in methanol (MeOH) (Honeywell, Charlotte, NC) and kept in the dark at −20 °C. The abbreviations of all compounds studied are summarized in Table SI-2. Ammonium hydroxide (NH_4_OH, ACS grade), potassium persulfate (K_2_S_2_O_8_, ACS grade), sodium hydroxide (NaOH, ACS grade), hydrochloric acid (HCl, ACS grade), and formic acid (LC-MS grade) were purchased from Fisher Scientific (Pittsburgh, PA). Domestic sludge reference material (NIST 2781) was obtained as our standard reference material (SRM). Ultrapure water was obtained from the Milli-Q (MQ) system (Millipore, Bedford, USA). Oasis weak anion exchange (WAX) cartridges (500 mg, 6 ml) were purchased from Waters (Milford, MA). Finally, 0.2-μm Captiva premium regenerated cellulose (RC) syringe filters were used for sample preparation (Agilent, USA) after the background interferences and sorption loss tests.

#### Sample extraction

Biosolids were prepared following EPA method 1633 with minor modifications. Briefly, wet/dry ratios were determined by drying 5 to 10 g of wet samples at 110 °C for at least 12 h, and all the PFAS concentrations reported in this study are on a dry weight (dw) basis. Based on the wet/dry ratio, wet biosolids (about 0.5 g in dw) were used for extraction. Samples were spiked with surrogate (SUR) mixture (0.5–10 ng) and extracted three times with 0.3% methanolic NH_4_OH using sequential extraction volumes of 10, 15, and 5 ml. Combined extracts were diluted, concentrated under nitrogen, brought to 50 ml and adjusted to pH 6.5 and 7 when necessary, before cleanup. Sample cleanup was performed with solid phase extraction (SPE) WAX cartridges. For samples in which cartridge clogged during loading, two WAX cartridges were used for the same sample and eluates were combined. The internal standard (IS) mixture was added to each sample elution to achieve a final concentration of 1 ng/ml. Spiked eluates were stored at 4 °C until further analysis.

#### TOP assay

The TOP assay was performed to measure the oxidizable precursors of PFAS not detected by direct measurement. Composite samples were created from California (*n* = 9), Kentucky (*n* = 3), and Arizona (*n* = 3), while only one subset of samples was taken from the other 12 states. The extraction procedure was conducted as described above, but without adding the surrogate mixture before the extraction. Surrogates were not added before oxidation because the alkaline persulfate reaction may oxidize or transform those compounds. After the extraction, 10 ml of each extract was transferred to a 15-ml centrifuge tube and blown down to complete dryness under a gentle flow of ultrapure N_2_. Aliquots of 5 ml of MQ water were added to the 15-ml tubes to reconstitute the extracts. To each tube, 135 mg K_2_S_2_O_8_ and 2.5 ml 1 N NaOH were added and the sealed samples were incubated for 10 h at 85 °C. The TOP assay reagent dose was selected based on previous laboratory experience with biosolids/sludge matrices. After the TOP assay, 5 ml of the sample was transferred to a 15-ml tube, and the sample was diluted with another 5 ml MQ. Samples were neutralized to pH 6 to 7 with 12 N HCl and 3% NH_4_OH. Surrogate mixture (0.5–10 ng) was spiked into each tube at this step, and samples went through the SPE procedure as described above.

#### PFAS analysis

Samples were analyzed on an Agilent 1290 Infinity liquid chromatograph coupled with an Agilent 6495B triple quadrupole mass spectrometer (LC-MS/MS). Mobile phase A was 5 mM of ammonium acetate in MQ water and 100% MeOH served as mobile phase B. The gradient started at 10% B and reached 100% B at 14.5 min with a flow rate at 0.4 ml/min. Five microliters of each sample was injected onto an Agilent Zobrax Eclipse Plus C18 analytical column (3.0 × 50 mm, 1.8 μm) preceded by an Agilent Zobrax Eclipse Plus C18 guard column (4.6 × 5 mm, 1.8 μm). A delayed column (Agilent Zobrax Eclipse Plus C18, 4.6 × 50 mm, 3.5 μm) was installed after the solvent mixer to prevent potential background PFAS from the mobile phases. The MS was operated in negative ionization mode. The detailed LC and MS parameters are listed in Table SI-3. The acquired data were processed by Agilent MassHunter Quantitative (B.09.00) analysis software to calculate concentration. The final concentrations were corrected by surrogates and internal standard recoveries.

#### Quality assurance and quality control (QA/QC)

The SRM was analyzed using the same extraction procedure used for the samples. Procedural blanks were prepared in every extraction batch. Sample duplicate, matrix spike (MS) and matrix spike duplicate (MSD) were performed for every 15 samples. The precision between samples and duplicates was calculated as relative percent difference (RPD), which was calculated using RPD % = $\frac{|\left(Csample-Cduplicate\right)|}{(Csample+Cduplicate)/2}\times 100$, where *C*_sample_ and *C*_duplicate_ are the concentration measured in the sample and in its duplicate.

### Data analysis

The studied PFAS were categorized into seven groups: (i) perfluoroalkyl carboxylic acids (PFCAs), including PFBA, PFPeA, PFHxA, PFHpA, PFOA, PFNA, PFDA, PFUnA, PFDoA, PFTrDA, and PFTeDA; (ii) perfluoroalkyl sulfonic acids (PFSAs) including PFBS, PFHpS, PFHxS, PFOS, PFNS, and PFDS; (iii) fluorotelomer sulfonic acids (FTSAs) including 6-2 FTS and 8-2 FTS; (iv) fluorotelomer carboxylic acids (FTCAs) including 3-3 FTCA, 5-3 FTCA, and 7-3 FTCA; (v) perfluorooctane sulfonamidoacetic acids (FOSAAs) including NMeFOSAA and NEtFOSAA; (vi) perfluorooctane sulfonamides (FOSA) including PFOSA; and (vii) perfluorooctane sulfonamide ethanols (FOSEs) including NMeFOSE and NEtFOSE.

## Results and discussion

### Method performance

**Fig. 1:**
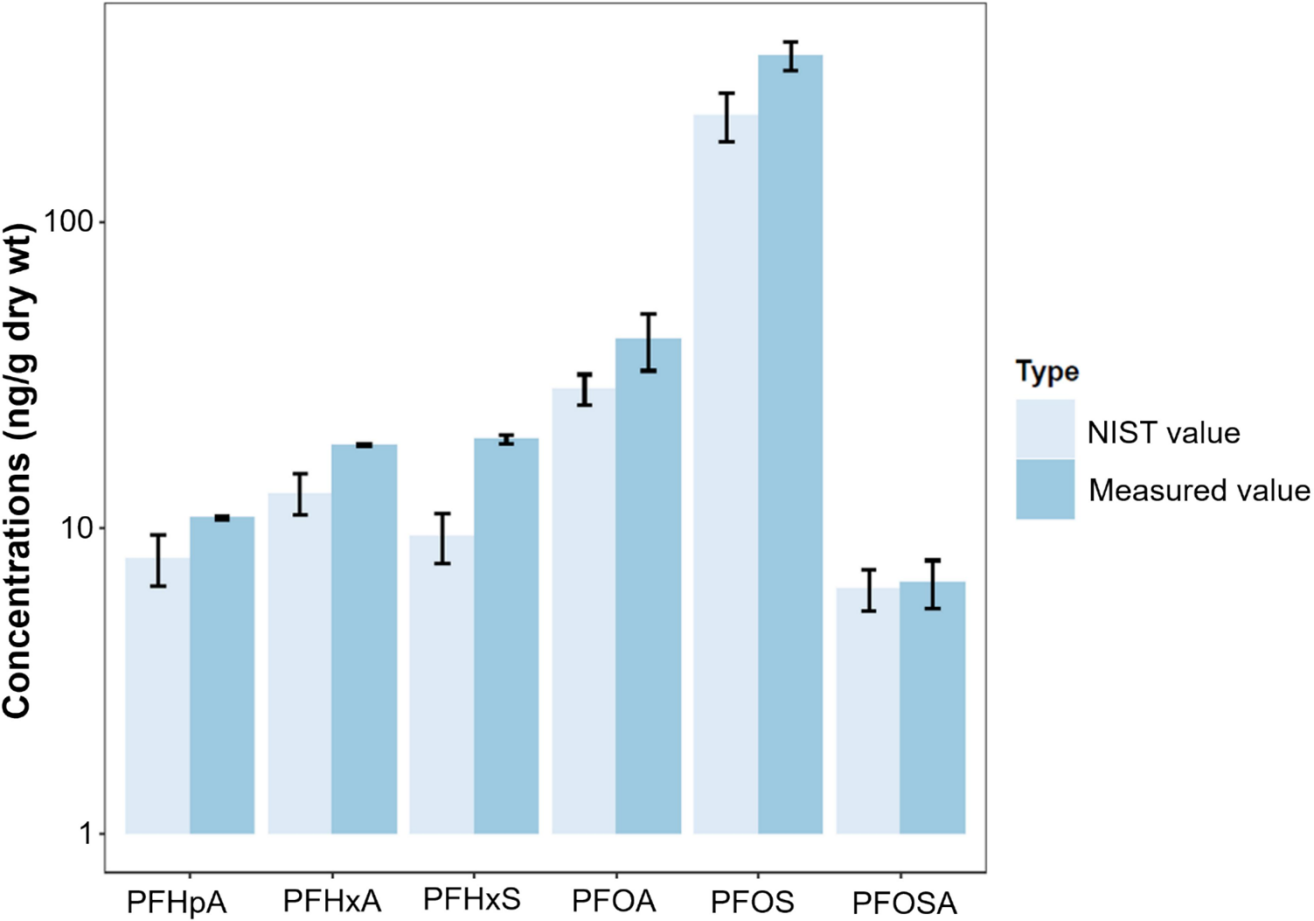
Comparison between measured concentrations and concentrations reported for the NIST 2781 certified reference material (CRM) for domestic sludge. Error bars represent uncertainties of the certified values and standard deviation (SD) for measured values

Comparisons were conducted between measured concentrations and NIST 2781 domestic sludge concentrations (Fig. 1). Difference between measured values and NIST values were 36% for PFHxA, 70% for PFHxS-total, 30% for PFHpA, 37% for PFOA, 44% for PFOS-total, and 5% for PFOSA. The larger discrepancy between the measured value and NIST value of PFHxS-total might be due to matrix induced signal enhancement during the LC-MS/MS analysis and heterogeneity of the sample. In addition, NIST values are non-certified reference values, which may also contribute to the observed difference. Surrogate recoveries ranged from 18 to 137% (Table SI-4). Most of the recoveries were within 70–130% except for NEtFOSE (d9-N-EtFOSE), NMeFOSE (d_7_-N-MeFOSE), PFTeDA (M2PFTeDA), and PFTrDA (average of M2PFDoA and M2PFTeDA) which showed lower recoveries (range 18–77%) and PFBA which had high recovery (range 94–137%). The instrument detection limits for various PFAS ranged between 0.009 to 0.1 ng/ml. Recovery rates from matrix spike experiments for the various analytes ranged between 41.7 and 132.6% in biosolids (Table SI-5). The RPD was within 30% for all the analytes except for PFNS (57.9% ± 15.9%) (Table SI-5). No lab contamination was observed in the reagent blanks and method blanks. Although the biosolid matrix varies widely including dewatered composite sludge, centrifuge cake, Class B biosolids, primary sludge, and activated sludge, NIST 2781 is the only commercially available reference material that provides values, though PFAS concentrations are not certified, and therefore, we used it to evaluate the method.

The complexity of the biosolid matrix may significantly affect the behavior of PFAS and can further impact analytical recovery, instrument response, and method performance. One recent study assessed the performance of EPA Method 1633 to evaluate PFAS in biosolids (Oza et al. 2025a). They compared the results among three laboratories while two of the labs followed EPA Method 1633. The authors reported significant difference in the measured PFAS concentrations between the labs and thus advocated for appropriate QA/QC protocols and certified materials to be used to validate the method. Our results agree with this observation. Although QA/QC can be modified specifically to the sludge/biosolid matrix, addressing the heterogeneity of the matrix is difficult and is not under the control of the testing laboratory. Sampling strategies for sewage sludge should be the focus of future work to capture representative samples from the facility. Compositing samples collected over several weeks/months can minimize such variance in the measurements. A similar approach was utilized to report baseline levels of hundreds of contaminants in nationally representative samples of biosolids in the past (McClellan and Halden 2010; Venkatesan and Halden 2013, 2014b).

### Occurrence of PFAS in US biosolids

**Fig. 2:**
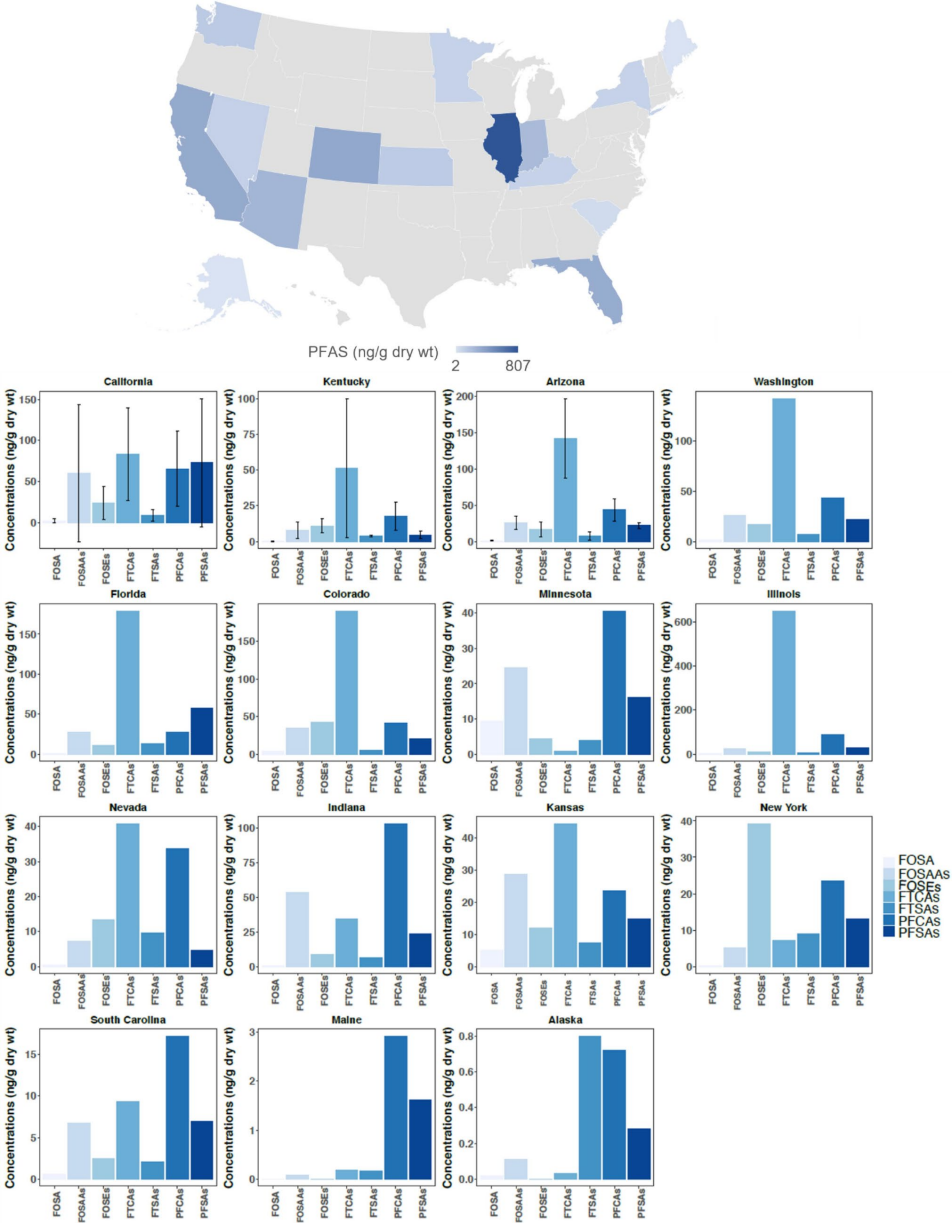
Concentration of PFAS detected in this study in biosolids from 15 US states. Each state only has one sample except for California (*n* = 9), Kentucky (*n* = 3), and Arizona (*n* = 3), error bars represent SD

**Fig. 3:**
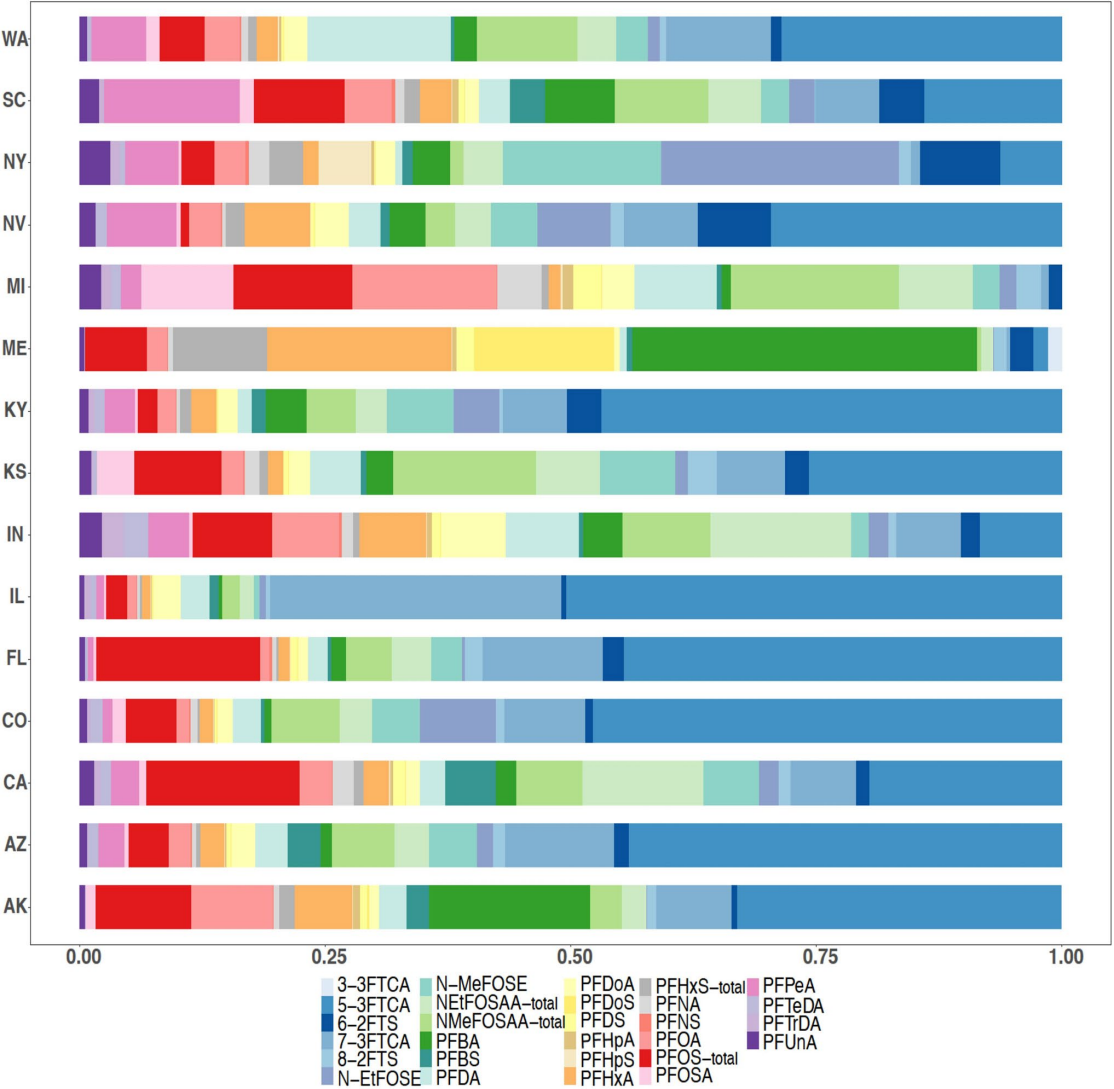
Contribution of each target compound to the totality of PFAS detected in biosolids. Individual PFAS concentrations are provided in Table SI-6

**Fig. 4:**
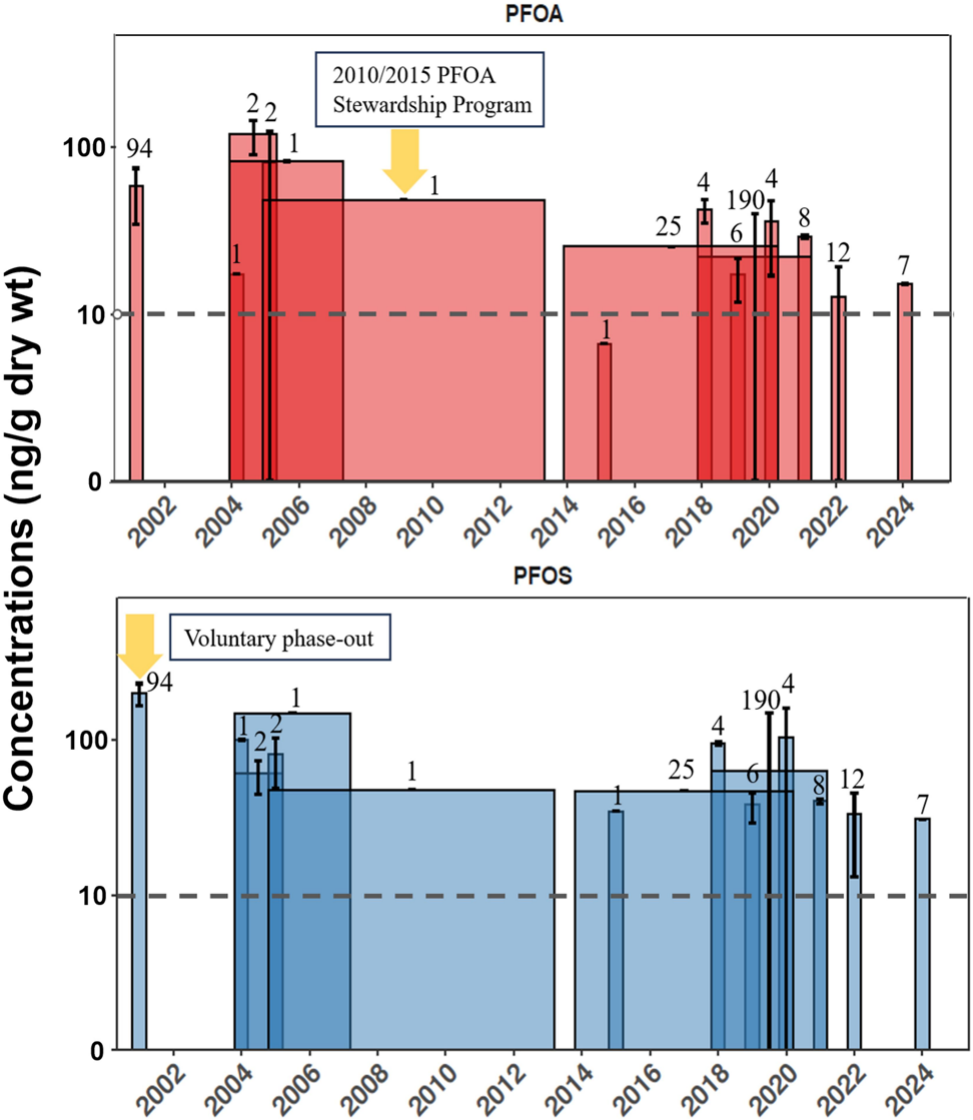
Change in average PFOA and PFOS concentrations over 25 years in US biosolids. Bars represent average concentrations reported in individual studies or datasets, and extended bars indicate studies in which sample collection spanned multiple years. Numbers above the bars indicate the number of WWTP datasets included in each study. The dashed line represents the EPA risk assessment level of 1 ng/g dry weight. Detailed numerical values and study information are provided in Table SI-7

Concentrations of PFAS in biosolids from different states are shown on Fig. 2. Twenty-eight out of 40 PFAS compounds were detected in the samples, with 18 compounds detected in all the biosolid samples analyzed in this study. The relative percentage of each target compound to total PFAS content in biosolids is shown in Fig. 3. The top five most abundant PFAS were 5-3 FTCA (70.2 ± 84.7 ng/g), 7-3 FTCA (23.7 ± 43.7 ng/g), PFOS (22.1 ± 45.2 ng/g), NEtFOSAA (17.4 ± 36.4 ng/g), and NMeFOSAA (14.2 ± 15.1 ng/g). Comparisons between this survey and the National Sewage Sludge Survey conducted previously are shown in Table 1 (Venkatesan and Halden 2013). The average concentrations of PFOS, PFDA, PFOA, PFDoA, PFUnA, and PFOSA significantly decreased compared to the previous survey. The samples for the previous national survey were collected in 2001, before the phasing out of long chain PFAS by several companies in the USA. This is clearly reflected in the concentration of abundant PFAS shifting to the shorter chain compounds, which were the replacement chemicals for the phased-out PFAS. Mean levels of PFOS and PFOA dropped by 95% and 81%, respectively, in samples analyzed in this study compared to the 2001 survey samples. PFAS levels in the present study also were comparable to other recent studies which quantified PFAS levels in biosolids collected in the USA and Canada (Table 1) (Loganathan et al. 2007; Sepulvado et al. 2011; Letcher et al. 2020; Lakshminarasimman et al. 2021; Caniglia et al. 2022; Gewurtz et al. 2024; Oza et al. 2025a). Although PFOA and PFOS levels have declined—an effect observed following their phase-outs in the early 2000s and 2010, respectively—their concentrations in recent samples remain approximately an order of magnitude higher than the EPA’s risk assessment level of 1 ng/g (Fig. 4).

**Table 1:** Concentrations of PFAS in biosolids in this study versus those reported in previous surveys

Compounds	This US study (2014-2020)		Previous US Survey (2001) (Venkatesan and Halden 2013)		% difference in mean levels between 2001 survey and this study	Other studies*
						Concentration (ng/g) (Min, Max)
	Concentration (ng/g) Avg (Min, Max)	Detection Frequency (%)	Concentration (ng/g) Avg (Min, Max)	Detection Frequency (%)		
5-3FTCA	70.2 (0.01, 407)	100				(3.1, 657.8)
7-3FTCA	23.7 (0.02, 239)	100				(2.6, 105.2)
PFOS-total	22.0 (0.13, 186)	100	403 (308, 618)	100	-95%	(0.49, 110)
NEtFOSAA-Total	17.3 (0.05, 191)	100				(2.1, 12)
NMeFOSAA-total	14.2 (0.02, 82.8)	100				(3.85, 16.89)
PFBS	10.2 (0.03, 110)	100	3.4 (2.5, 4.8)	60	200%	(0.14, 25)
N-MeFOSE	9.52 (0.0, 50.4)	62				(0, 100.8)
PFDA	7.22 (0.04, 29.4)	100	26.1 (6.9, 59.1)	100	-72%	(0.986, 53)
PFOA	6.46 (0.11, 47.8)	100	34 (11.8, 70.3)	100	-81%	(0.211, 219)
PFPeA	6.36 (0.00, 20.3)	90	3.5 (1.8, 6.7)	100	81%	(0, 14)
PFHxA	5.67 (0.12, 15.8)	100	6.2 (2.5, 11.7)	100	-8%	(0, 18.1)
PFBA	4.53 (0.32, 27.1)	100	2 (1.2, 3.2)	80	127%	(0, 49)
PFDoA	4.51 (0.02, 22.6)	100	10.9 (4.5, 26)	100	-58%	(0.3, 10)
6-2FTS	3.73 (0.01, 13.3)	100				(4.2, 37)
PFNA	3.11 (0.01, 25.3)	100	9.2 (3.2, 21.1)	100	-66%	(0.42, 20)
PFUnA	2.79 (0.00, 13.3)	97	11.7 (2.8, 38.7)	100	-76%	(0.397, 12)
N-EtFOSE	2.65 (0.00, 23.5)	24				(3.7, 31)
8-2FTS	2.54 (0.00, 13.3)	97				(4.2, 37)
PFTeDA	1.96 (0.00, 17.2)	86				(0.05, 12)
PFHxS-total	1.79 (0.03, 5.93)	100	5.9 (7.4, 5.4)	100	-69%	(0.966, 12)
PFDS	1.74 (0.00, 19.5)	90				(0.11, 426)
PFOSA	1.62 (0.00, 9.26)	97	20.7 (2.2, 68.1)	100	-92%	(0, 5.7)
PFTrDA	1.25 (0.00, 11.0)	69				(0.0382, 12)
PFHpA	0.59 (0.00, 4.11)	76	3.4 (1.2, 11.7)	80	-82%	(0.08, 5.2)
PFHpS	0.27 (0.00, 5.14)	41				(0, 12)
PFNS	0.17 (0.00, 0.71)	86				(0, 5.3)
PFDoS	0.02 (0.00, 0.70)	3				(0, 7.8)
3-3FTCA	0.003 (0.00,0.07)	3				(0, 12)

Loganathan et al. 2007; Sepulvado et al. 2011; Letcher et al. 2020; Lakshminarasimman et al. 2021; Caniglia et al. 2022; Gewurtz et al. 2024; Oza et al. 2025a

For the 15 US states we evaluated, samples from Illinois showed most noticeable total detected PFAS levels with concentration peaks observed for 5:3 FTCA (408 ng/g) and 7:3 FTCA (240 ng/g). This may be because the corresponding WWTP serves a population of 2,200,000 and processes domestic, commercial, and industrial wastewater. Samples from Maine and Alaska featured lower levels of PFAS. The observation for Maine in this study was surprising, given that the state recently banned the land application of biosolids due to high contamination of agricultural areas that received biosolids. However, the Maine WWTP from which the samples were collected, only covers a population of 3,100 and operates at a relatively small-scale with possibly limited industrial inputs; thus, it may not well represent the overall PFAS biosolids trend in larger settlements with industrial inputs or for the state. Overall, FTCAs and PFCAs were the dominant PFAS in most of the states. Recent studies from the USA and Canada also reported high concentrations of 5:3 FTCA, the dominant PFAS in biosolids, along with 7:3 FTCA (Schaefer et al. 2023; Gewurtz et al. 2024; Oza et al. 2025a). 5:3 FTCA is a short-chain precursor as well as the degradation product of other PFAS precursors (O’Connor et al. 2022). Multiple studies reported that degradation of 6:2 fluorotelomer substances can yield the formation of 5:3 FTCA (Zhang et al. 2016, 2021; Field and Seow 2017; Hamid et al. 2020; Gonzalez et al. 2021). 5:3 FTCA is also widely used in carpets, textiles, and food packaging leading to frequent detection in landfill leachates (Coffin et al. 2023; Schwartz-Narbonne et al. 2023; Capozzi et al. 2023). One study demonstrated the release of 5:3 FTCA from carpets in a model anaerobic landfill reactor (Lang et al. 2016), suggesting that it is likely released from residences and laundry facilities to WWTPs during washing of textiles and carpets. 5:3 FTCA is not only the intermediate compound in the environment, surprisingly, it is also a metabolic intermediate in humans. One study reported 5:3 FTCA and 7:3 FTCA in ski wax technicians’ blood samples after exposure to high levels of fluorotelomer alcohol in the air (Nilsson et al. 2013). At varied rates, 5:3 FTCA can further transform to short-chain PFCAs such as PFBA, PFPeA, and PFHxA (Wang et al. 2012; Maldonado et al. 2022; Schaefer et al. 2023).

**Fig. 5:**
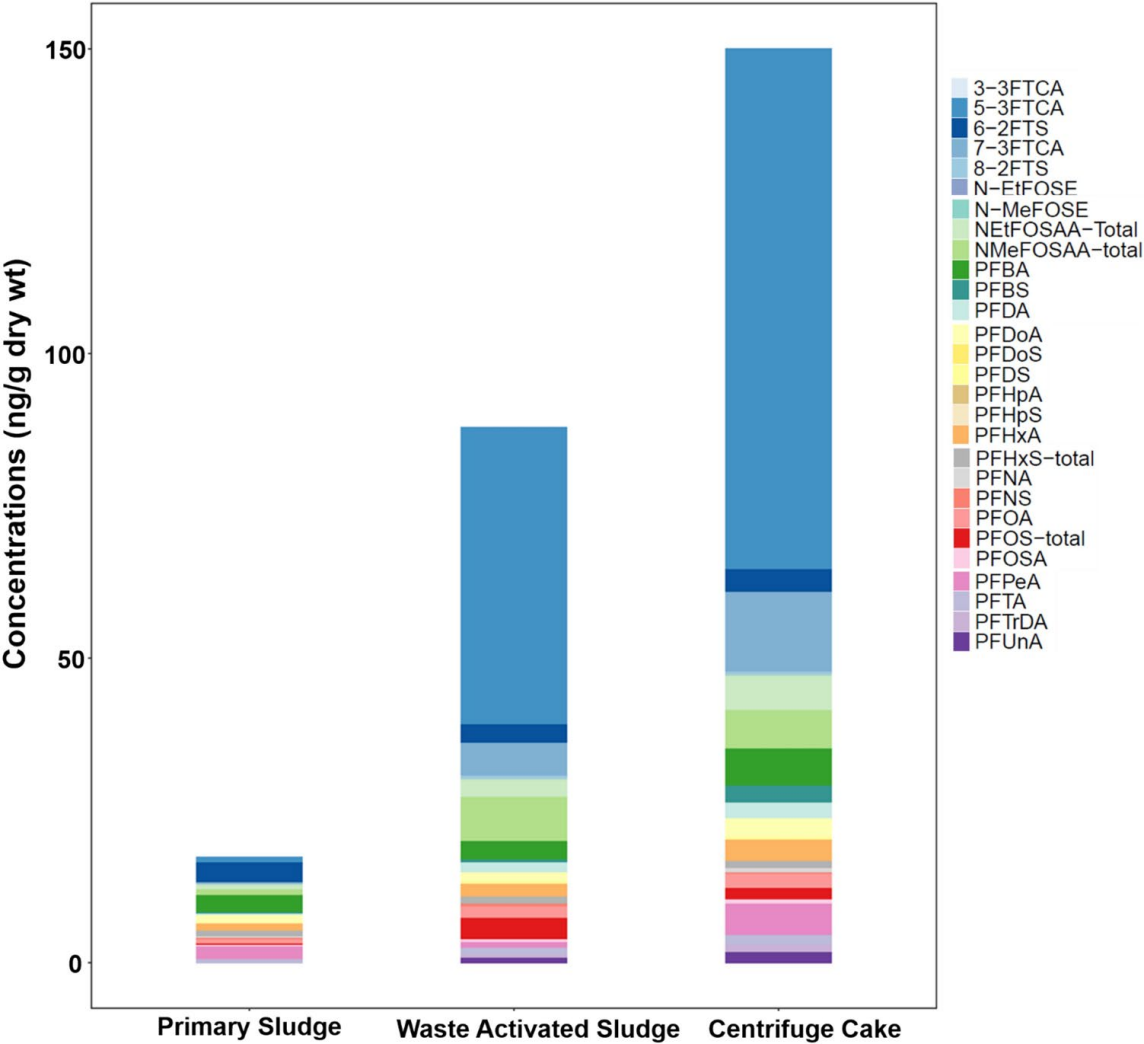
PFAS levels in Kentucky biosolid samples from different WW treatment stages

Biosolids from Kentucky also observed 5:3 FTCA to be predominant. From the same WWTP, samples from three different wastewater treatment stages: primary sludge, waste activated sludge, and centrifuge cake were analyzed. The total PFAS concentrations increased from 17.3 ng/g (primary sludge) to 150 ng/g (centrifuge cake) (Fig. 5), with 5:3 FTCA significantly increasing from 0.9 ng/g to 85.4 ng/g. Similarly, a study by Oza et al. (2025b) observed pre- and post-stabilized sludge products over 27 water resource recovery facilities and found the 5:3 FTCA values to be higher in the post-stabilized samples, where formation was attributed to the anaerobic degradation of 6:2 fluorotelomer alcohols. These observations of higher 5:3 FTCA concentrations were consistent with other studies which stated that the terminal end products of 6:2 FTS and 6:2 FTOH comprise FTCAs, including 5:3 FTCA, which further show resistance to biological transformation (Fredriksson et al. 2022; Oza et al. 2025b). Therefore, an increase in 5:3 FTCA can be due to (i) degradation or transformation during biological treatment such as activated sludge or biological filtration; (ii) sludge treatment like anaerobic digestion, thermal drying, stabilization or centrifugation which may further concentrate 5:3 FTCA in biosolids; or (iii) resistance to degradation to shorter-chain PFAS by biological transformation (Kabadi et al. 2020; Oza et al. 2025a, b). Furthermore, samples collected from different treatment stages may have entered the facility at different times, and temporal variability in the influent PFAS concentrations may also contribute to the observed differences. Thus time-resolved sampling would be needed to directly evaluate PFAS transformation and changes through the plant.

### Total oxidizable precursors of PFAS in US biosolids

**Fig. 6:**
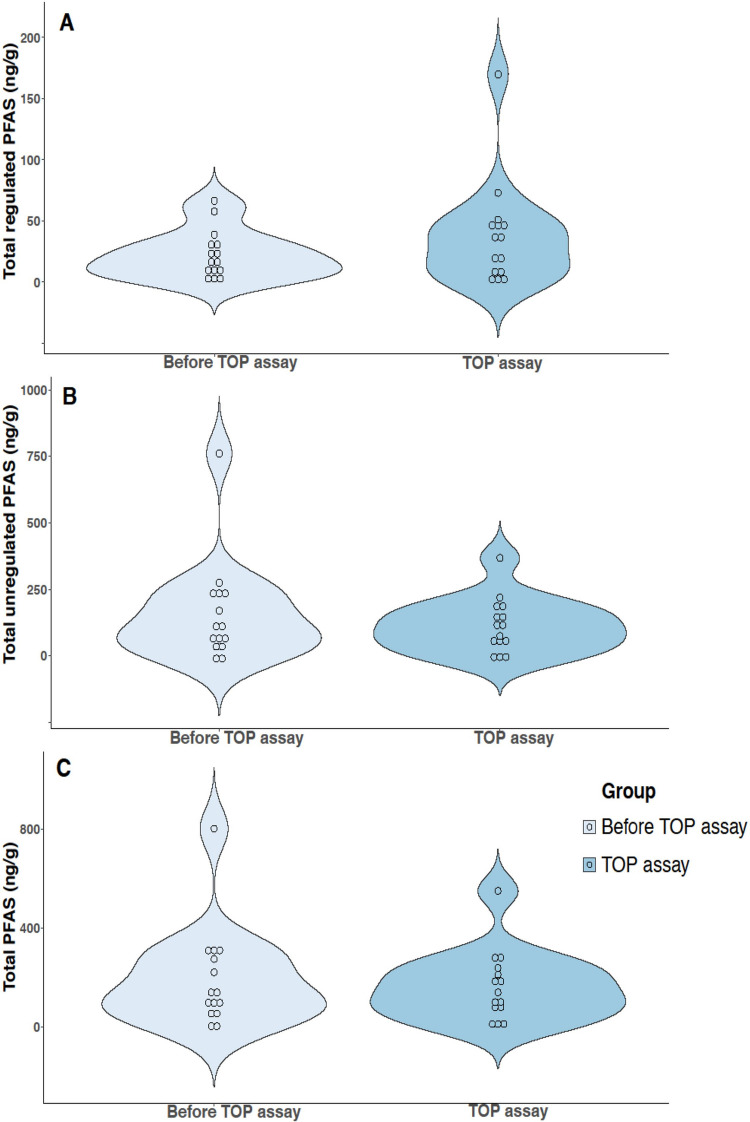
Distribution of total PFAS concentrations before and after the TOP assay. Violin plots show the sample-level distribution of summed concentrations for **A** regulated PFAS (PFHxS, PFOA, PFOS, and PFNA), **B** unregulated PFAS, and **C** all detected PFAS. Open circles represent individual biosolid samples. The TOP assay results reflect PFAS concentrations measured after oxidative conversion of precursors. Compound-specific changes are shown in Figure SI-1

In the TOP assay, PFAA precursors are chemically oxidized to yield PFCAs of related chain lengths. The difference between the amount of PFCAs before (i.e., via target analysis) and after the TOP assay can provide information on the quantity of “oxidizable” precursors in a sample. However, it needs to be noted that the oxidative processes simulated in the TOP assay are not exactly identical to those in the environmental degradation of precursors and the assay may not cover all types of PFAS. Furthermore, the TOP assay results should be interpreted semi-quantitatively because precursor oxidation depends on the reaction conditions and sample matrix. Biosolids and sludge are complex, heterogeneous matrices that may consume oxidant and reduce oxidation efficiency. Therefore, although the reagent dose was selected based on prior laboratory experience, complete oxidation of all PFAS precursors cannot be assumed, and direct quantitative comparison with studies using different protocols or matrices should be made cautiously. In this study, application of the TOP assay to the composite samples from 15 US states produced a *C*_4_–*C*_12_ PFAAs as well as some precursors. Interestingly, the presence of precursors even after oxidation indicates both incomplete oxidation and the complexity of the samples. As can be seen in Fig. 6, Panel A, with respect to regulated PFAS, it is observed that prior to the TOP assay, most samples have low concentrations (ranging between 0 and 50 ng/g), with a median value around 25–30 ng/g. However, following the TOP assay, the distribution shifts slightly upwards, with the median concentration visibly higher than the “Before” group. Hence, the marked increase in concentrations from pre- to post-TOP assay suggests the presence of unknown oxidizable precursors that are transformed into detectable regulated PFAS. Similarly, with respect to total unregulated PFAS (ng/g), before the TOP assay, many samples had median PFAS concentration ranging from 100 to 150 ng/g and after the TOP assay, the distribution appears to be somewhat narrower with the median concentration similar to before assay. This indicates that there are transformations of non-target precursors into measurable unregulated PFAS, albeit at lower levels than the regulated PFAS. Consequently, the total PFAS concentrations post-TOP assay (Fig. 6C) shows an upward shift in the median concentration but a decrease in the maximum concentration observed. This observation is consistent with other studies that have reported increases in terminal PFAS in biosolids after the oxidation assay (Kim et al. 2022). When examining the individual PFAS detected in the biosolid composites, several PFAS that serve as precursors**,** such as fluorotelomer compounds (FTCAs) and sulfonamide-based compounds (N-MeFOSE, NEtFOSAA-L, NMeFOSAA) (Figure SI-1, right side panel), showed decreases in their relative concentrations (indicated as negative % change). In agreement with this, several of the stable oxidation products such as PFCA (e.g., PFNA, PFTA) and PFSA (e.g., PFNS, PFOSA) (Figure SI-1, left side panel) saw an increase in their concentration (indicated as positive % change) (Kim et al. 2022). This conclusively shows that biosolids could serve as a repository of several unmonitored precursors and intermediaries (in addition to terminal PFAS) which can be transformed into stable end-products either during the chemical treatment processes or during biotransformation when repurposed for agricultural or land-based use. This was also observed in a mesocosm study with PFAS concentration increasing over the course of 3 years (Venkatesan and Halden 2014a).

Changes in branched-to-linear isomer ratio in biosolids relative to influent wastewater may provide insight into PFAS transformation, sorption behavior, and partitioning during wastewater treatment processes. Understanding isomer distributions can therefore improve source identification and predictions of PFAS fate, mobility, and bioavailability following land application of biosolids. When it comes to branched/linear isomer ratios for PFHxS and PFOS, in general, the linear form was dominant in biosolids. However, shifts in the composition of PFHxS and PFOS isomers were observed after the TOP assay (Figure SI-2). Branched/linear ratios overall decreased for PFHxS after the TOP assay except for samples from CO, KY, NV, and WA, while for half of the samples branched/linear ratios decreased for PFOS after the TOP assay. For those with increased PFHxS ratios, either both branched and linear isomer concentrations increased but branched isomer increased more after TOP assay (samples from CO and WA), or branched isomers increased while linear isomers decreased (samples from KY and NV). For those samples with increased PFOS ratios, either both branched and linear isomers increased (CO, IN, and KY), or branched isomer increased while linear isomer decreased (samples from AK and IL), or branched isomer stayed the same while linear isomer decreased (samples from MI and ME).

In samples from AK, the branched isomers dominated, but precursors seem to largely produce linear PFHxS, hence the decrease in branched/linear ratios after the TOP assay. Samples from states like KY or WA might have precursors that form more branched PFHxS, as suggested by increased concentrations after the TOP assay. This highlights regional differences in PFHxS sources and precursor composition. Linear PFOS is observed to be the dominant form in most regions. Overall, in the locations studied here, PFOS was found to be less affected by precursor transformation as compared to PFHxS.

Changes in branched/linear ratios should be interpreted cautiously. The pre-oxidation concentrations were measured from the targeted PFAS workflow, whereas post-oxidation concentrations were measured from the TOP assay workflow. Therefore, the differences in branched/linear ratios may reflect not only precursor oxidation but also matrix effects, oxidation efficiency, extraction efficiency, and potential isomer-specific mechanisms of precursor transformation pathways. Although these results were not used to infer specific mechanisms of precursor transformation, further studies would be needed to evaluate isomer-specific precursor sources, transformation, and fate in biosolids, since understanding branched and linear distribution can aid in the effective management of biosolids after land application.

### Study limitations

Although this study quantified 40 PFAS compounds, this number represents only a small fraction of the many thousands of PFAS that are likely present in biosolids. Broader screening for total organic fluorine, untargeted, or suspected compounds could help identify additional PFAS and precursors that are not captured by targeted methods. For the TOP assay, composite samples were created for three states (CA, KY, and AZ), and such mixing may introduce variability in sample analyses compared to the other states (with one sample). Furthermore, surrogates were not added before TOP oxidation, the post-oxidation concentrations were not corrected by recovery. This approach avoids potential oxidation or transformation of surrogates during TOP reaction, but it also limits direct quantitative comparison between pre-and post-oxidation results in our study. The difference between pre-and post-oxidation concentrations may reflect not only precursor oxidation, but also sample heterogeneity, matrix effects, extraction efficiency, oxidation efficiency, and lack of surrogate correction in the TOP assay. In addition, the samples analyzed in this study were collected over a prolonged period (2014–2020), with most of samples were collected in 2015. While terminal PFAS are highly stable, long-term storage could allow slow transformation of precursors, potentially influencing observed concentrations. Furthermore, the complex chemical composition of biosolids poses additional challenges, as the oxidant can be consumed by organic matter or other constituents of the matrix, limiting the production of hydroxyl radicals and reducing precursor oxidation efficiency. This matrix effect renders incomplete precursor conversion, suggesting that the TOP assay may require further optimization (conditions such as oxidant dose, reaction time, and temperature) for different biosolids types. Lastly, the TOP assay results provide evidence for the presence of oxidizable PFAS precursors in biosolids. Although changes in linear and branched isomer profiles were observed before and after oxidation, these patterns should not be overinterpreted as direct evidence of isomer-specific transformation pathways because TOP assay results may be affected by oxidation efficiency, matrix effects, and method-specific limitations.

As reported in this study, PFAS distribution is highly variable across different states, within individual states, and among treatment processes. The levels are also influenced by site-specific characteristics of wastewater sources. Since we analyzed only one sample from several US states, the measured concentrations may not reflect the PFAS trends observed across the state, nor fully capture the extent of PFAS contamination in biosolids. In our previous work, we addressed this limitation by creating composite samples of biosolids from within regions to provide baseline contaminant levels in US biosolids (Venkatesan and Halden 2014b). In this study, to evaluate the variance within a single state, we selected CA and compared PFAS levels from nine different locations (Figure SI-3, Table SI-8). The concentrations were highly variable within the state for almost all detected PFAS, although 5:3 FTCA consistently dominated the samples, consistent with observations across other states. The standard deviations reported in Table SI-8 can serve as a useful guide to understand the expected distribution across other states and to estimate the uncertainty in the baseline PFAS levels and trends reported in this and similar surveys.

## Conclusions

In the 2014–2020 samples examined here, PFOS is no longer the most prevalent congener, and PFOA also did not dominate in the samples. Instead, 5:3 FTCA and 7:3 FTCA emerged as the most abundant compounds across the majority of biosolid samples, reflecting changes in PFAS usage patterns and highlighting the growing importance of fluorotelomer-based precursors. Although PFOA and PFOS are not dominant and decrease in concentration over time (Fig. 4), their levels remain above 1 ng/g, which is considered a significant risk according to the recently published US EPA risk assessment document (EPA 2025). Since PFOA and PFOS account for only a small fraction of the total PFAS in biosolids, this raises important questions about the risks associated with PFAS mixtures and their application on land as a result of biosolids recycling. Changes in PFAS profiles after the TOP assay underscore the role of precursor transformation. Our study findings support concerns raised in the literature over the environmental and human health risks associated with land application of biosolids and emphasize the need for continued monitoring, further investigation into PFAS transformation pathways, and optimization of oxidation-based methods specific to the biosolid matrix.

## Supplementary Information

Below is the link to the electronic supplementary material.

ESM 1 (DOCX 344 KB)

## Data Availability

The authors declare that the data supporting the findings of this study are available within the paper and its Supplementary Information files. Should any raw data files be needed in another format, they are available from the corresponding author upon reasonable request. Data on the locations and specific treatment processes of wastewater treatment plants are anonymized and not available due to agreements with participating facilities.
