# Supplementary material for: Occurrence of per- and polyfluoroalkyl substances (PFAS) and their precursors in biosolids across 15 U.S. states
Source: Environ Sci Pollut Res Int. 2026 Jul 21;33(24):12290–304. doi: 10.1007/s11356-026-38066-3 (PMC13469210; doi:10.1007/s11356-026-38066-3)
Supplement: Supplementary file 1 [file 11356_2026_38066_MOESM1_ESM.docx]

**Supplementary Material**

**Occurrence of Per- and Polyfluoroalkyl Substances (PFAS) and Their Precursors in Biosolids Across 15 U.S. States**

Xiayan Ye^1^, Amith Maroli^2^, Santhoshi Chitthaluri^3^, Erin M. Driver^4^, Rolf U. Halden^4^, Arjun K. Venkatesan^3^

^1^ Cleveland Clinic Research, Cleveland Clinic, Cleveland, OH, 44195, USA

^2^ Department of Environmental Medicine and Climate Science, Icahn School of Medicine at Mount Sinai, New York, NY, 10029, USA

^3^ Department of Civil and Environmental Engineering, New Jersey Institute of Technology, Newark, New Jersey, 07102, USA

^4^ Center for Environmental Health Engineering, The Biodesign Institute, Arizona State University, Tempe, AZ 85281, USA

^*^Corresponding author email: [arjun.venkatesan@njit.edu](mailto:arjun.venkatesan@njit.edu); phone: +1 973-596-5884

**Table SI-1: Information on sample and processing facilities**

| **No.** | **State/ Location** | **Year** | **Population** | **Sample and Facility Information** |
| --- | --- | --- | --- | --- |
| 1 | CA | 2015 | 130,000 | Biosolids. |
|  |  |  |  | Domestic, commercial and industrial inputs. The Discharger thickens primary sludge with gravity thickeners and waste-activated sludge through dissolved air flotation, treats the thickened sludge with anaerobic digestion, dewaters the digested sludge with a centrifuge. |
| 2 | CA | 2015 | 50,000 | Dewatered Composite. |
|  |  |  |  | Domestic, commercial and industrial inputs. Sludge is anaerobically digested, dewatered using a belt filter press, and air dried. |
| 3 | WA | 2020 | 38,000 | Biosolids. |
|  |  |  |  | Domestic, commercial and industrial inputs. One significant industrial user manufactures laser cutting equipment; activated sludge plant. |
| 4 | CA | 2015 | 97,000 | Biosolids from Drying Beds. |
|  |  |  |  | Domestic, commercial and industrial input. Sludge is anaerobically digested, dewatered using a belt filter press, then air-dried to produce biosolids. |
| 5 | CA | 2015 | 1,400,000 | Domestic, commercial and industrial inputs. Residential and main connections to businesses and industries: food service, automotive, metal finishing, photo processing, and manufacturing. Anaerobic digester tanks. After about 30 days, the sludge is transferred to open lagoons to dry. |
| 6 | FL | 2016 | 2,300,000 | Class B Biosolids. |
|  |  |  |  | Domestic, commercial and industrial inputs. Anaerobically digested to meet Class B biosolids stabilization criteria, dewatered by centrifuges, and trucked to land application or landfill sites. |
| 7 | CA | 2016 | 4,000,000 | Biosolids. |
|  |  |  |  | Domestic, commercial and industrial inputs. Solid fractions recovered from wastewater treatment processes include grit, primary screenings, primary sludge and skimming, thickened waste activated sludge, digested sludge screenings and digester cleaning solids. The fine solids (grit, primary screenings, digested sludge screenings, digester cleaning solids) that consist of primarily inorganic materials are hauled away to landfills. The remaining solid fractions (primary sludge and skimming, thickened waste activated sludge) are anaerobically digested. The digested solids are screened and dewatered using centrifuges. Thermophilic digestion is used to generate Class A “EQ” biosolids using anaerobic digesters. |
| 8 | CO | 2015 | 211,000 | Biosolids. |
|  |  |  |  | Domestic, commercial and industrial inputs. 110,000 domestic + 101,000 employees in commercial and industrial businesses. |
| 9 | MI | 2015 | 125,000 | Grab sample. |
|  |  |  |  | 72% domestic, 28% industrial inputs. |
| 10 | CA | 2015 | 230,000 | Dewatered sludge cake. |
|  |  |  |  | Domestic (60%), commercial (30%) and industrial (10%) wastewater inputs. |
| 11 | CA | 2015 | 740,000 | Biosolids. |
|  |  |  |  | Domestic, commercial and industrial inputs. Produces Class B biosolids using an anaerobic digestion process, during which the sludge is subjected to high temperatures (95°F to 131°F) for at least 15 days. |
| 12 | CA | 2015 | 485,000 | Biosolids. |
|  |  |  |  | Domestic, commercial and industrial inputs. |
| 13 | IL | 2015 | 2,200,000 | Biosolids. |
|  |  |  |  | Domestic, commercial and industrial inputs. |
| 14 | NV | 2016 | 970,000 | Biosolids. |
| 15 | CA | 2015 | 12,000 people (population equivalent) | Biosolids. |
|  |  |  |  | 35% industrial; Sanitary Plant includes a collection system and treats sanitary wastewater from airplanes and airport facilities, including terminal restrooms, hangars, restaurants, and shops. The Industrial Plant includes a separate collection system and treats industrial wastewater from maintenance shops and vehicle washing, as well as first-flush stormwater runoff from industrial areas. Process drains in the wastewater treatment plant area route to the Sanitary Plant, which may collect stormwater, overflow industrial wastewater from the equalization tank, and other incidental process wastewater. |
| 16 | AZ | 2015 | 120,000 | Biosolids. |
|  |  |  |  | Domestic, commercial and industrial inputs. Sludge undergoes thickening for anaerobic digestion followed by dewatering and Class B pathogen reduction with a minimum of 38 percent reduction in volatile solids for land application. |
| 17 | AZ | 2017 | 74,000 | Dewatered Sludge. |
|  |  |  |  | Domestic, commercial and industrial inputs. The solids from the primary and secondary systems are pumped to the anaerobic digester for final solids treatment. This process breaks down the organic material and generates natural gas (methane) which is used to produce electricity for the facility. The solids are then disposed of in the bio-solids disposal area. |
| 18 | KY | 2020 | 751,000 | Primary sludge, waste activated sludge, and centrifuge cake samples. |
| 19 | KY | 2020 | 751,000 | Domestic, commercial and industrial inputs. Processes biosolids from other KY treatment plants. Primary solids are anaerobically digested then combined with thickened waste-activated sludge for dewatering and drying. Digester gas provides much of the energy needed for drying, and waste heat from drying helps heat the digesters. |
| 20 | KY | 2020 | 751,000 |  |
| 21 | IN | 2017 | 44,000 | Dewatered sludge. |
|  |  |  |  | 54% domestic, 28% industrial, 18% other; WAS storage and includes coarse bubble diffused aeration to prevent the WAS from becoming septic and keep the tanks well mixed before dewatering. WAS from the ASH tank is dewatered using a centrifuge process. |
| 22 | KS | 2016 | 77,484 | Biosolids. |
|  |  |  |  | Domestic, commercial and industrial inputs. |
| 23 | NY | 2015 | 53,000 | Sludge composite. |
|  |  |  |  | Domestic, commercial and industrial inputs. |
| 24 | AZ | 2014 | 2,000,000 | Sludge sample. |
| 25 | SC | 2020 | 100,000 | - |
| 26 | AK | 2015 | 8,000 | Biosolids |
|  |  |  |  | No significant industrial users. |
| 27 | ME | 2016 | 3,100 | Liquid Biosolids. |
|  |  |  |  | Class B land application was abundant in Maine in decades past but has been diminishing since the late 2000s. Class B biosolids are usually used at dairy farms for hay and feed corn crops. Lime stabilized biosolids. |

**Table SI-2. PFAS native compounds, surrogates, and internal standards used in this study**

| **#** | **Compound name** | **Type** | **Abbreviation** |
| --- | --- | --- | --- |
| n1 | Nonafluoro-3,6-dioxaheptanoic acid | native | NFDHA |
| n2 | Perfluorobutanoic acid | native | PFBA |
| n3 | Perfluoro-3-methoxypropanoic acid | native | PFMPA |
| n4 | 3-Perfluoropropyl propanoic acid | native | 3:3FTCA |
| n5 | Perfluoropentanoic acid | native | PFPeA |
| n6 | Perfluoro-4-methoxybutanoic acid | native | PFMBA |
| n7 | Hexafluoropropylene oxide dimer acid | native | HFPO-DA |
| n8 | Perfluorobutanesulfonic acid | native | PFBS |
| n9 | Perfluorohexanoic acid | native | PFHxA |
| n10 | Perfluoro(2-ethoxyethane)sulfonic acid | native | PFEESA |
| n11 | 1H,1H, 2H, 2H-Perfluorohexane sulfonic acid | native | 4:2FTS |
| n12 | 2H,2H,3H,3H-Perfluorooctanoic acid | native | 5:3FTCA |
| n13 | Perfluoropentanesulfonic acid | native | PFPeS |
| n14 | Perfluoroheptanoic acid | native | PFHpA |
| n15 | 4,8-dioxa-3H-perfluorononanoic acid | native | ADONA |
| n16 | Perfluorohexanesulfonic acid | native | PFHxS |
| n17 | Perfluorooctanoic acid | native | PFOA |
| n18 | 1H,1H, 2H, 2H-Perfluorooctane sulfonic acid | native | 6:2FTS |
| n19 | 3-Perfluoroheptyl propanoic acid | native | 7:3FTCA |
| n20 | Perfluoroheptanesulfonic acid | native | PFHpS |
| n21 | Perfluorononanoic acid | native | PFNA |
| n22 | Perfluorooctanesulfonamide | native | PFOSA |
| n23 | Perfluorooctanesulfonic acid | native | PFOS |
| n24 | N-methyl perfluorooctanesulfonamide | native | NMeFOSA |
| n25 | Perfluorodecanoic acid | native | PFDA |
| n26 | N-ethyl perfluorooctanesulfonamide | native | NEtFOSA |
| n27 | 1H,1H, 2H, 2H-Perfluorodecane sulfonic acid | native | 8:2FTS |
| n28 | 9-chlorohexadecafluoro-3-oxanone-1-sulfonic acid | native | 9Cl-PF3ONS |
| n29 | Perfluorononanesulfonic acid | native | PFNS |
| n30 | Perfluoroundecanoic acid | native | PFUnA |
| n31 | N-methyl perfluorooctanesulfonamidoacetic acid | native | NMeFOSAA |
| n32 | N-ethyl perfluorooctanesulfonamidoacetic acid | native | NEtFOSAA |
| n33 | Perfluorodecanesulfonic acid | native | PFDS |
| n34 | Perfluorododecanoic acid | native | PFDoA |
| n35 | N-methyl perfluorooctanesulfonamidoethanol | native | NMeFOSE |
| n36 | N-ethyl perfluorooctanesulfonamidoethanol | native | NEtFOSE |
| n37 | 11-chloroeicosafluoro-3-oxaundecane-1-sulfonic acid | native | 11Cl-PF3OUdS |
| n38 | Perfluorotridecanoic acid | native | PFTrDA |
| n39 | Perfluorododecanesulfonic acid | native | PFDoS |
| n40 | Perfluorotetradecanoic acid | native | PFTA / PFTeDA |
| s1 | Perfluoro-n-[1,2,3,4-^13^C_4_]butanoic acid | SUR | MPFBA |
| s2 | Perfluoro-n-[1,2,3,4,5-^13^C5]pentanoic acid | SUR | M5PFPeA |
| s3 | 2,3,3,3-Tetrafluoro-2-(1,1,2,2,3,3,3-heptafluoropropoxy-^13^C_3_-propanoic acid | SUR | M3HFPO-DA |
| s4 | Sodium perfluoro-1-[2,3,4-^13^C_3_]butanesulfonate | SUR | M3PFBS |
| s5 | Perfluoro-n-[1,2,3,4,6-^13^C5]hexanoic acid | SUR | M5PFHxA |
| s6 | Sodium 1H,1H,2H,2H-perfluoro-1-[1,2-^13^C_2_]hexane sulfonate | SUR | M2-4:2FTS |
| s7 | Perfluoro-n-[1,2,3,4-^13^C_4_]heptanoic acid | SUR | M4PFHpA |
| s8 | Sodium perfluoro-1-[1,2,3-^13^C_3_]hexanesulfonate | SUR | M3PFHxS |
| s9 | Perfluoro-n-[^13^C_8_]octanoic acid | SUR | M8PFOA |
| s10 | Sodium 1H,1H,2H,2H-perfluoro-1-[1,2-^13^C_2_]-octane sulfonate | SUR | M2-6:2FTS |
| s11 | Perfluoro-n-[^13^C_9_]nonanoic acid | SUR | M9PFNA |
| s12 | Perfluoro-1-[^13^C_8_]octanesulfonamide | SUR | M8FOSA |
| s13 | Sodium perfluoro-[^13^C_8_]octanesulfonate | SUR | M8PFOS |
| s14 | N-methyl-d_3_-perfluoro-1-octanesulfonamide | SUR | d3-N-MeFOSA |
| s15 | Perfluoro-n-[1,2,3,4,5,6-^13^C_6_]decanoic acid | SUR | M6PFDA |
| s16 | Sodium 1H,1H,2H,2H-perfluoro-1-[1,2-^13^C_2_]-decane sulfonate | SUR | M2-8:2FTS |
| s17 | N-ethyl-d_5_-perfluoro-1-octanesulfonamide | SUR | d5-N-EtFOSA |
| s18 | Perfluoro-n-[1,2,3,4,5,6,7-^13^C_7_]undecanoic acid | SUR | M7PFUnA |
| s19 | N-deuteriomethylperfluoro-1-octanesulfonamidoacetic acid | SUR | d3-N-MeFOSAA |
| s20 | N-deuterioethylperfluoro-1-octanesulfonamidoacetic acid | SUR | d5-N-EtFOSAA |
| s21 | Perfluoro-n-[1,2-^13^C_2_]dodecanoic acid | SUR | MPFDoA |
| s22 | N-methyl-d_7_-perfluorooctanesulfonamidoethanol | SUR | d7-N-MeFOSE |
| s23 | N-ethyl-d_9_-perfluorooctanesulfonamidoethanol | SUR | d9-N-EtFOSE |
| s24 | Perfluoro-n-[1,2-^13^C_2_]tetradecanoic acid | SUR | M2PFTeDA |
| i1 | Perfluoro-n-[2,3,4-^13^C_3_]butanoic acid | IS | M3PFBA |
| i2 | Perfluoro-n-[1,2-^13^C_2_]hexanoic acid | IS | MPFHxA |
| i3 | Perfluoro-1-hexane[^18^O_2_]sulfonic acid | IS | MPFHxS |
| i4 | Perfluoro-n-[1,2,3,4-^13^C_4_]octanoic acid | IS | MPFOA |
| i5 | Perfluoro-n-[1,2,3,4,5-^13^C_5_] nonanoic acid | IS | MPFNA |
| i6 | Sodium perfluoro-1-[1,2,3,4-^13^C_4_]octanesulfonate | IS | MPFOS |
| i7 | Perfluoro-n-[1,2-^13^C_2_]decanoic acid | IS | MPFDA |

**Table SI-3. LC-MS/MS configuration and parameters**

(A) **LC parameters**

| **LC parameter** | **Value** |
| --- | --- |
| LC | Agilent G7120A 1290 Binary Pump |
|  | Agilent G7116A 1260 Multicolumn Thermostat |
|  | Agilent G7167A 1260 Multisampler |
| Analytical column | Agilent ZOBRAX Eclipse Plus C18 3.0 x 50 mm, 1.8 µm |
| Delayed column | Agilent ZOBRAX Eclipse Plus C18 4.6 x 50 mm, 3.5 µm |
| Column temperature | 50 °C |
| Injection volume | 5 µL |
| Mobile phase | A) 5 mM Ammonium acetate in water B) 100% MeOH |
| Flow rate | 0.4 mL/min |
| Gradient | Time (min) %B |
|  | 0.0 10 |
|  | 0.5 10 |
|  | 2.0 30 |
|  | 14.0 95 |
|  | 14.5 100 |
| Stop time | 16.5 minutes |
| Post time | 6 minutes |

(B) **MS parameters**

| **MS Parameter** | | **Value** |
| --- | --- | --- |
| MS | Agilent 6495 Triple Quadrupole MS/MS Agilent Jet Stream ESI source | |
| Gas Temperature | 175 °C | |
| Gas flow | 17 L/min | |
| Nebulizer | 20 psi | |
| Sheath gas temperature | 275 °C | |
| Sheath gas flow | 11 L/min | |
| Capillary voltage (Neg) | 2500 V | |
| Nozzle voltage (Neg) | 0 V | |
| iFunnel  High pressure RF (Neg) Low pressure RF (Neg) | 90 V 40 V | |

**Table SI-4. SUR recoveries for each compound**

|  | Mean (%) | | | SD (%) |
| --- | --- | --- | --- | --- |
| MPFBA | | 133.12 | | 24.61 |
| M5PFPeA | | | 76.16 | 11.44 |
| M3PFBS | | | 82.26 | 13.10 |
| M5PFHxA | | | 78.06 | 13.19 |
| M3PFHxS | | | 78.29 | 10.78 |
| M4PFHpA | | | 80.45 | 12.77 |
| M3-6-2FTS | | | 103.06 | 46.16 |
| M8PFOA | | | 78.25 | 11.94 |
| M8PFOS | | | 112.68 | 38.20 |
| M9PFNA | | | 88.59 | 12.76 |
| M5PFHxA | | | 78.06 | 13.19 |
| M2-8-2FTS | | | 105.10 | 45.18 |
| M6PFDA | | | 77.30 | 13.33 |
| d3-N-MeFOSAA | | | 79.85 | 17.63 |
| d5-N-EtFOSAA | | | 86.01 | 22.53 |
| M7PFUnA | | | 74.25 | 15.90 |
| M8FOSA | | | 62.68 | 16.69 |
| M2PFDoA | | | 60.05 | 18.77 |
| Average (M2PFDoA, M2PFTeDA) | | | 46.28 | 16.72 |
| M2PFTeDA | | | 28.92 | 22.66 |
| d7-N-MeFOSE | | | 30.25 | 18.11 |
| d9-N-EtFOSE | | | 16.82 | 11.32 |

**Table SI-5. Relative percent difference and spike recovery**

|  | **RPD (%)** | |  | **Spike recovery (%)** | |
| --- | --- | --- | --- | --- | --- |
|  | Mean | SD |  | Mean | SD |
| PFBA | 22.70 | 5.38 |  | 128.88 | 54.40 |
| 3-3FTCA | NA | NA |  | 99.14 | 26.96 |
| PFPeA | 8.22 | 3.14 |  | 111.15 | 70.00 |
| PFBS | 12.40 | 5.04 |  | 76.80 | 5.56 |
| PFHxA | 11.80 | 8.79 |  | 88.70 | 1.85 |
| PFHxS | 10.40 | 6.81 |  | 96.13 | 19.76 |
| PFHpA | 28.00 | 11.60 |  | 91.77 | 5.92 |
| 5-3 FTCA | 10.50 | 0.10 |  | 85.86 | 33.71 |
| 6-2 FTS | 20.30 | 8.05 |  | 97.55 | 3.31 |
| PFOA | 10.20 | 5.15 |  | 114.83 | 30.82 |
| PFOS | 10.40 | 1.55 |  | 127.45 | 25.75 |
| PFNA | 4.60 | 3.17 |  | 104.59 | 22.60 |
| 7-3 FTCA | 5.30 | 0.17 |  | 41.73 | 11.25 |
| PFNS | 57.90 | 15.90 |  | 68.84 | 17.60 |
| 8-2 FTS | 6.80 | 5.32 |  | 70.17 | 2.90 |
| PFDA | 7.88 | 7.68 |  | 67.97 | 72.38 |
| NMeFOSAA | 28.10 | 6.28 |  | 128.24 | 13.70 |
| NEtFOSAA | 15.20 | 9.45 |  | 100.76 | 33.56 |
| PFDS | 15.70 | 22.20 |  | 132.61 | 21.79 |
| PFUnA | 22.50 | 4.86 |  | 72.87 | 15.20 |
| PFOSA | 10.20 | 4.41 |  | 86.34 | 20.18 |
| PFDoA | 10.10 | 14.10 |  | 100.90 | 26.66 |
| PFDoS | NA | NA |  | 106.29 | 30.12 |
| PFTrDA | 16.00 | 13.80 |  | 93.75 | 6.89 |
| PFTeDA | 7.10 | 10.10 |  | 88.78 | 12.63 |
| NMeFOSE | 9.41 | 13.30 |  | 95.93 | 17.62 |
| NEtFOSE | NA | NA |  | 94.95 | 15.55 |

**Table SI-6: PFAS concentrations (ng/g dry wt) from each state**

| ng/g dry wt | CA | KY | AZ | WA | FL | CO | MI | IL | NV | IN | KS | NY | SC | ME | AK |
| --- | --- | --- | --- | --- | --- | --- | --- | --- | --- | --- | --- | --- | --- | --- | --- |
| PFBA | 6.68 | 4.06 | 3.12 | 3.42 | 5.03 | 2.64 | 1.04 | 3.45 | 4.09 | 9.40 | 3.81 | 3.76 | 3.24 | 1.75 | 0.32 |
| 3-3FTCA | 0.00 | 0.00 | 0.00 | 0.00 | 0.00 | 0.00 | 0.00 | 0.00 | 0.00 | 0.00 | 0.00 | 0.00 | 0.00 | 0.07 | 0.00 |
| PFPeA | 9.37 | 3.03 | 6.87 | 8.19 | 2.10 | 3.72 | 2.10 | 7.19 | 7.90 | 9.74 | 0.00 | 5.37 | 6.30 | 0.00 | 0.00 |
| PFBS | 16.17 | 1.35 | 8.70 | 0.51 | 1.14 | 1.35 | 0.46 | 7.62 | 1.03 | 0.95 | 0.76 | 1.05 | 1.61 | 0.03 | 0.04 |
| PFHxA | 8.38 | 2.53 | 6.32 | 3.17 | 3.71 | 4.96 | 1.33 | 7.53 | 7.34 | 15.77 | 2.26 | 1.61 | 1.51 | 0.93 | 0.12 |
| PFHxS-total | 3.05 | 1.09 | 1.17 | 1.34 | 0.75 | 0.72 | 0.69 | 1.71 | 2.11 | 1.45 | 1.10 | 3.36 | 0.72 | 0.48 | 0.03 |
| PFHpA | 1.07 | 0.00 | 0.60 | 0.35 | 0.00 | 0.46 | 1.17 | 0.95 | 0.00 | 1.32 | 0.00 | 0.35 | 0.32 | 0.03 | 0.02 |
| 5-3FTCA | 61.84 | 45.06 | 113.70 | 41.57 | 140.16 | 161.85 | 0.01 | 407.77 | 32.56 | 19.30 | 35.12 | 6.18 | 6.39 | 0.07 | 0.65 |
| 6-2FTS | 4.32 | 3.36 | 3.87 | 1.58 | 6.83 | 2.57 | 1.34 | 3.81 | 8.18 | 4.49 | 3.32 | 7.92 | 2.05 | 0.12 | 0.01 |
| PFOA | 10.43 | 1.85 | 5.75 | 5.27 | 3.26 | 4.63 | 14.63 | 7.72 | 3.60 | 15.78 | 3.11 | 3.17 | 2.20 | 0.11 | 0.16 |
| PFHpS | 0.22 | 0.00 | 0.00 | 0.14 | 0.31 | 0.00 | 0.12 | 0.00 | 0.14 | 0.00 | 0.00 | 5.14 | 0.00 | 0.00 | 0.00 |
| PFOS-total | 49.33 | 1.93 | 10.63 | 6.71 | 52.34 | 17.52 | 12.09 | 17.42 | 0.95 | 18.66 | 12.09 | 3.28 | 4.19 | 0.31 | 0.19 |
| PFNA | 6.77 | 0.33 | 1.14 | 1.05 | 1.30 | 2.41 | 4.49 | 2.41 | 0.42 | 2.75 | 2.09 | 2.05 | 0.41 | 0.02 | 0.01 |
| 7-3FTCA | 21.03 | 6.23 | 28.63 | 15.45 | 38.35 | 28.03 | 0.79 | 239.85 | 8.24 | 15.16 | 9.37 | 0.95 | 2.98 | 0.02 | 0.15 |
| PFNS | 0.20 | 0.02 | 0.23 | 0.05 | 0.71 | 0.18 | 0.01 | 0.08 | 0.07 | 0.46 | 0.08 | 0.23 | 0.14 | 0.00 | 0.00 |
| 8-2FTS | 3.95 | 0.35 | 3.28 | 1.00 | 5.67 | 2.93 | 2.47 | 3.30 | 1.47 | 1.74 | 4.09 | 1.15 | 0.02 | 0.06 | 0.02 |
| PFDA | 8.29 | 1.40 | 8.54 | 21.23 | 6.36 | 9.70 | 8.39 | 23.32 | 3.55 | 17.16 | 6.97 | 0.68 | 1.46 | 0.04 | 0.06 |
| NMeFOSAA-total | 21.28 | 4.81 | 16.45 | 14.93 | 14.61 | 23.47 | 17.00 | 14.56 | 3.28 | 20.62 | 19.81 | 1.35 | 4.33 | 0.02 | 0.06 |
| NEtFOSAA-total | 38.78 | 3.06 | 9.12 | 5.76 | 12.46 | 11.24 | 7.47 | 11.74 | 3.98 | 32.86 | 8.91 | 3.90 | 2.44 | 0.06 | 0.05 |
| PFDS | 3.88 | 0.12 | 1.20 | 0.35 | 2.10 | 0.73 | 2.79 | 1.00 | 0.34 | 1.99 | 0.72 | 0.08 | 0.24 | 0.09 | 0.01 |
| PFUnA | 4.67 | 0.91 | 2.08 | 1.19 | 1.94 | 2.79 | 2.19 | 4.37 | 1.80 | 5.34 | 1.66 | 3.10 | 0.93 | 0.02 | 0.01 |
| PFOSA | 2.00 | 0.18 | 0.98 | 1.92 | 0.58 | 4.34 | 9.26 | 0.97 | 0.39 | 0.63 | 5.18 | 0.17 | 0.60 | 0.00 | 0.02 |
| PFDoA | 4.47 | 1.87 | 6.15 | 3.43 | 3.17 | 5.27 | 3.27 | 22.63 | 3.80 | 15.25 | 2.92 | 2.00 | 0.64 | 0.03 | 0.02 |
| PFDoS | 0.00 | 0.00 | 0.00 | 0.00 | 0.00 | 0.00 | 0.00 | 0.00 | 0.00 | 0.00 | 0.00 | 0.00 | 0.00 | 0.70 | 0.00 |
| PFTrDA | 2.03 | 0.56 | 0.43 | 0.00 | 0.00 | 1.02 | 1.00 | 4.74 | 0.00 | 5.12 | 0.25 | 0.99 | 0.19 | 0.00 | 0.00 |
| PFTeDA | 3.17 | 0.97 | 2.39 | 0.48 | 0.60 | 3.91 | 0.96 | 4.30 | 1.18 | 5.53 | 0.42 | 0.42 | 0.00 | 0.00 | 0.00 |
| N-MeFOSE | 18.00 | 6.55 | 12.51 | 4.66 | 10.02 | 16.41 | 2.67 | 4.23 | 5.19 | 4.24 | 10.40 | 15.70 | 1.31 | 0.00 | 0.00 |
| N-EtFOSE | 5.93 | 4.36 | 3.98 | 1.63 | 0.68 | 25.87 | 1.69 | 4.79 | 8.13 | 4.48 | 1.59 | 23.52 | 1.12 | 0.00 | 0.00 |

**Table SI-7: PFOA and PFOS concentrations over 25 years in U.S. biosolids.**

| **Sampling period** | **No of WWTPs** | **PFOA (ng/g) *** | **PFOS (ng/g) *** | **Reference** |
| --- | --- | --- | --- | --- |
| 2001 | 94 | 34±22 | 403±127 | (Venkatesan and Halden 2013) |
| 2004 | 1 | <3 | 100 (81-160) | (Schultz et al. 2006) |
| 2004-2005 | 1 | 144±63 | 37±17 | (Sinclair and Kannan 2006) |
|  | 1 | 25±12 | 70±29 |  |
| 2005 | 1 | 65.83±88.54 | 64.1±40.7 | (Loganathan et al. 2007) |
|  | 1 | 17.2±12.6 | 42.5±7.5 |  |
| 2004-2007 | 1 | (8-68) | (80-219) | (Sepulvado et al. 2011) |
| 2005-2013 | 1 | 23.5 (BDL-601) | 22.5 (BDL-68.1) | (Armstrong et al. 2016) |
| 2015 | 1 | 0.44 | 12 | (Johnson 2022) |
| 2018 | 4 | 4.34±5.64 | 8.37±4.69 | (Kim Lazcano et al. 2019) |
| 2018 | 36 | (BDL-18) | (3-90) | (2020) |
| 2019 | 6 | 3.03±1.63 | 14.64±6.03 | (Tavasoli 2020) |
| 2020 | 1 | 0.211 | 9.4 | (Caniglia et al. 2022) |
|  | 4 | 12.75±9.86 | 107.5±147.46 | (Dorin Bogdan and Christopher Curran 2021) |
|  | 7 | 2.86±2.14 | 32.65±45.81 | (Schaefer et al. 2022) |
|  | 1 | 0.3±0 | 27.75±8.61 | (Pepper et al. 2021) |
| 2021 | 8 | 8.5±0.3 | 16.1±1.2 | (Thompson et al. 2023) |
| 2022 | 12 | 1.6±2.1 | 11.2±9.51 | (Oza et al. 2025) |
| 2018-2021 | 190 | 4.8±11 | 40±179 | (Link et al. 2024) |
| 2024 | 7 | 2.3 | 9.5 | (Rahman et al. 2025) |
| 2014-2020 | 25 | 6.46 (0.11-47.8) | 22 (0.13-186) | This study |

*Values presented either as mean ± standard deviation data or as range given as (min-max); BDL: Below Detection Limit

**Table SI-8. PFAS concentrations from nine CA samples.**

| PFAS (ng/g dry wt) | Min | Max | Average | SD |
| --- | --- | --- | --- | --- |
| PFBA | 1.48 | 27.13 | 6.68 | 7.91 |
| PFPeA | 1.86 | 20.29 | 9.37 | 6.15 |
| PFBS | 0.52 | 101.76 | 16.17 | 32.35 |
| PFHxA | 3.79 | 13.25 | 8.38 | 3.17 |
| PFHxS-total | 0.46 | 5.93 | 3.05 | 1.61 |
| PFHpA | 0.00 | 4.11 | 1.07 | 1.27 |
| 5-3FTCA | 3.49 | 145.18 | 61.84 | 50.16 |
| 6-2FTS | 1.19 | 13.26 | 4.32 | 3.53 |
| PFOA | 2.50 | 47.84 | 10.43 | 14.28 |
| PFOS-total | 1.36 | 186.73 | 49.33 | 74.69 |
| PFHpS | 0.00 | 0.78 | 0.22 | 0.26 |
| PFNA | 0.23 | 25.34 | 6.77 | 8.71 |
| 7-3FTCA | 2.58 | 43.30 | 21.03 | 12.25 |
| PFNS | 0.00 | 0.41 | 0.20 | 0.15 |
| 8-2FTS | 0.81 | 12.64 | 3.95 | 3.76 |
| PFDA | 0.70 | 29.35 | 8.29 | 8.64 |
| NMeFOSAA-total | 7.64 | 82.78 | 21.28 | 23.54 |
| NEtFOSAA-total | 7.38 | 191.23 | 38.78 | 61.38 |
| PFDS | 0.17 | 19.54 | 3.88 | 6.34 |
| PFUnA | 0.54 | 13.34 | 4.67 | 4.33 |
| PFOSA | 0.31 | 8.35 | 2.00 | 2.50 |
| PFDoA | 1.51 | 14.94 | 4.47 | 4.30 |
| PFTrDA | 0.00 | 11.01 | 2.03 | 3.57 |
| PFTA | 0.30 | 17.23 | 3.19 | 5.36 |
| N-MeFOSE | 0.00 | 50.38 | 17.40 | 19.02 |
| N-EtFOSE | 0.00 | 17.06 | 4.48 | 7.25 |

**Figure SI-1.** Average changes for each PFAS after the TOP assay. Error bar represents SD.

**Figure SI-2.** PFHxS and PFOS branched/linear ratios before and after the TOP assay shown by state.

**Figure SI-3.** Variance in PFAS concentrations measured in nine biosolids samples collected from different locations across California.

**References:**

Armstrong DL, Lozano N, Rice CP, Ramirez M, Torrents A (2016) Temporal trends of perfluoroalkyl substances in limed biosolids from a large municipal water resource recovery facility. Journal of Environmental Management 165:88–95. https://doi.org/10.1016/j.jenvman.2015.09.023

Caniglia J, Snow DD, Messer T, Bartelt-Hunt S (2022) Extraction, analysis, and occurrence of per- and polyfluoroalkyl substances (PFAS) in wastewater and after municipal biosolids land application to determine agricultural loading. Front Water 4. https://doi.org/10.3389/frwa.2022.892451

Dorin Bogdan, Christopher Curran (2021) Evaluation of PFAS in wastewater treatment plants (WWTPs) across the United States. AECOM

Johnson GR (2022) PFAS in soil and groundwater following historical land application of biosolids. Water Research 211:118035. https://doi.org/10.1016/j.watres.2021.118035

Kim Lazcano R, de Perre C, Mashtare ML, Lee LS (2019) Per- and polyfluoroalkyl substances in commercially available biosolid-based products: The effect of treatment processes. Water Environment Research 91:1669–1677

Link GW, Reeves DM, Cassidy DP, Coffin ES (2024) Per- and polyfluoroalkyl substances (PFAS) in final treated solids (Biosolids) from 190 Michigan wastewater treatment plants. J Hazard Mater 463:132734. https://doi.org/10.1016/j.jhazmat.2023.132734

Loganathan BG, Sajwan KS, Sinclair E, Senthil Kumar K, Kannan K (2007) Perfluoroalkyl sulfonates and perfluorocarboxylates in two wastewater treatment facilities in Kentucky and Georgia. Water Research 41(20):4611–4620. https://doi.org/10.1016/j.watres.2007.06.045

Oza S, Bell KY, Xu Z, Wang Y, Wells MJM, Norton Jr. JW, Winchell LJ, Huang Q, Li H (2025) Surveillance of PFAS in sludge and biosolids at 12 water resource recovery facilities. Journal of Environmental Quality 54(1):6–19. https://doi.org/10.1002/jeq2.20595

Pepper IL, Brusseau ML, Prevatt FJ, Escobar BA (2021) Incidence of Pfas in soil following long-term application of class B biosolids. Science of The Total Environment 793:148449. https://doi.org/10.1016/j.scitotenv.2021.148449

Rahman A, Grieco S, Bahman B, Friedenthal A, White A, Williams T (2025) Understanding dynamics of PFAS in biosolids processed through composting, thermal drying and high temperature pyrolysis. Journal of Water Process Engineering 72:107508. https://doi.org/10.1016/j.jwpe.2025.107508

Schaefer CE, Hooper J, Modiri-Gharehveran M, Drennan DM, Beecher N, Lee L (2022) Release of poly- and perfluoroalkyl substances from finished biosolids in soil mesocosms. Water Res 217:118405. https://doi.org/10.1016/j.watres.2022.118405

Schultz MM, Higgins CP, Huset CA, Luthy RG, Barofsky DF, Field JA (2006) Fluorochemical Mass Flows in a Municipal Wastewater Treatment Facility. Environ Sci Technol 40(23):7350–7357. https://doi.org/10.1021/es061025m

Sepulvado JG, Blaine AC, Hundal LS, Higgins CP (2011) Occurrence and fate of perfluorochemicals in soil following the land application of municipal biosolids. Environ Sci Technol 45(19):8106–8112. https://doi.org/10.1021/es103903d

Sinclair E, Kannan K (2006) Mass Loading and Fate of Perfluoroalkyl Surfactants in Wastewater Treatment Plants. Environ Sci Technol 40(5):1408–1414. https://doi.org/10.1021/es051798v

Tavasoli E (2020) Distribution and Fate of Per- and Polyfluorinated Alkyl Substances (PFAS) in Wastewater Treatment Plants Discharging to Great Bay. Master’s Theses and Capstones

Thompson JT, Robey NM, Tolaymat TM, Bowden JA, Solo-Gabriele HM, Townsend TG (2023) Underestimation of Per- and Polyfluoroalkyl Substances in Biosolids: Precursor Transformation During Conventional Treatment. Environmental Science and Technology 57:3825–3832

Venkatesan AK, Halden RU (2013) National inventory of perfluoroalkyl substances in archived U.S. biosolids from the 2001 EPA National Sewage Sludge Survey. J Hazard Mater 0:413–418. https://doi.org/10.1016/j.jhazmat.2013.03.016

(2020) Initiatives to Evaluate the Presence of PFAS in Municipal Wastewater and Associated Residuals (Sludge/Biosolids) in Michigan. https://www.michigan.gov/-/media/Project/Websites/egle/Documents/Programs/WRD/IPP/pfas-initiatives-wastewater-sludge.pdf?rev=2f47b34f32804b349dcf219fec460ec5. Accessed 6 Oct 2025
